# Effects of Yeast Species and Processing on Intestinal Health and Transcriptomic Profiles of Atlantic Salmon (*Salmo salar*) Fed Soybean Meal-Based Diets in Seawater

**DOI:** 10.3390/ijms23031675

**Published:** 2022-01-31

**Authors:** Jeleel O. Agboola, Dominic D. Mensah, Jon Ø. Hansen, David Lapeña, Liv T. Mydland, Magnus Ø. Arntzen, Svein J. Horn, Ove Øyås, Charles McL. Press, Margareth Øverland

**Affiliations:** 1 Department of Animal and Aquacultural Sciences, Norwegian University of Life Sciences, P.O. Box 5003, NO-1432 Ås, Norway; dominic.duncan.mensah@nmbu.no (D.D.M.); jon.hansen@nmbu.no (J.Ø.H.); liv.mydland@nmbu.no (L.T.M.); ove.oyas@nmbu.no (O.Ø.); 2 Faculty of Chemistry, Biotechnology and Food Science, Norwegian University of Life Sciences, P.O. Box 5003, NO-1432 Ås, Norway; davidlapego@gmail.com (D.L.); magnus.arntzen@nmbu.no (M.Ø.A.); svein.horn@nmbu.no (S.J.H.); 3 Department of Preclinical Sciences and Pathology, Faculty of Veterinary Medicine, Norwegian University of Life Sciences, P.O. Box 5003, NO-1432 Ås, Norway; charles.press@nmbu.no

**Keywords:** *Cyberlindnera jadinii*, *Wickerhamomyces anomalus*, intestinal health, SBMIE, transcriptomics, distal intestine, spleen, autolysis

## Abstract

The objective of the current study was to examine the effects of yeasts on intestinal health and transcriptomic profiles from the distal intestine and spleen tissue of Atlantic salmon fed SBM-based diets in seawater. *Cyberlindnera jadinii* (CJ) and *Wickerhamomyces anomalus* (WA) yeasts were heat-inactivated with spray-drying (ICJ and IWA) or autolyzed at 50 °C for 16 h (ACJ and AWA), followed by spray-drying. Six diets were formulated, one based on fishmeal (FM), a challenging diet with 30% soybean meal (SBM) and four other diets containing 30% SBM and 10% of each of the four yeast fractions (i.e., ICJ, ACJ, IWA and AWA). The inclusion of CJ yeasts reduced the loss of enterocyte supranuclear vacuolization and reduced the population of CD8α labeled cells present in the lamina propria of fish fed the SBM diet. The CJ yeasts controlled the inflammatory responses of fish fed SBM through up-regulation of pathways related to wound healing and taurine metabolism. The WA yeasts dampened the inflammatory profile of fish fed SBM through down-regulation of pathways related to toll-like receptor signaling, C-lectin receptor, cytokine receptor and signal transduction. This study suggests that the yeast species, *Cyberlindnera jadinii* and *Wickerhamomyces anomalus* are novel high-quality protein sources with health-beneficial effects in terms of reducing inflammation associated with feeding plant-based diets to Atlantic salmon.

## 1. Introduction

In recent decades, the composition of Atlantic salmon (*Salmo salar*) diets has changed towards the use of more plant ingredients [1] due to limited availability and increased market prices of fishmeal (FM) [2]. Currently, commercial salmon diets contain about 75% plant-derived ingredients [1]. Soybean meal (SBM) is an attractive plant ingredient due to its availability and high protein content as well as its low production cost [3]. It has already been described that SBM contains anti-nutritional factors (ANFs), especially saponin, which can induce inflammation in the distal intestine (DI) of Atlantic salmon, a condition commonly referred to as SBM-induced enteritis (SBMIE) [4,5]. Dietary inclusion of SBM induced both local and systemic responses in Atlantic salmon [6,7]. Studies have shown up-regulation of genes associated with increased gut permeability in Atlantic salmon fed SBM-based diets [6,8], which may lead to translocation of opportunistic bacteria to the underlying mucosa [9,10]. Thus, the inclusion of SBM in salmon diets can have adverse effects on growth performance and fish health [11]. For these reasons, soy-protein concentrate, which is a refined soy product with low levels of ANFs, is currently used in commercial salmon diets. However, a recent study has shown that DI inflammation is still being observed in commercial salmon production in Norway [12].

Yeasts are gaining increasing interest as alternative ingredients for salmonids [12,13,14]. Yeasts are not only high-quality protein ingredients but also contain bioactive components that have the potential to mitigate SBMIE in Atlantic salmon [15,16,17]. The yeast cell wall contains bioactive components, including β-glucan, mannan and chitin, which has immune-modulating properties that reduce inflammation caused by SBM [15]. However, the bioactivity of these cell wall components depends on the yeast species, the fermentation conditions during yeast production and the downstream processing used before incorporating them into a salmon diet [13,18]. The inclusion of yeast in salmon diets could be a nutritional strategy to improve intestinal health and develop robust fish when feeding them with plant-based diets. Therefore, the objective of this study was to investigate the effect of yeasts on intestinal health and transcriptomic profiles of DI and spleen tissues from Atlantic salmon fed SBM-based diet in seawater. The applied yeasts, *Cyberlindnera jadinii* (CJ) and *Wickerhamomyces anomalus* (WA), were produced from wood sugars using in-house bioreactors. These yeasts were selected based on their functional effects reported in our previous study [15].

## 2. Results

### 2.1. Yeast Compositions

Yeast contained between 42 and 47% crude protein (Table 1). Autolysis increased the crude lipid content by 96% (from 2.9 to 5.7% DM) and 46% (from 2.8 to 4.1% DM) for CJ and WA, respectively (Table 1). Ash, total phosphorus, and gross energy contents of the two yeast species were similar and unaffected by the autolytic process (Table 1). The β-glucan, mannan and chitin content (% DM) of the inactivated yeast species were in the ranges of 15–16%, 8–11% and 0.3–0.5%. In both CJ and WA, autolysis reduced the content of β-glucan and mannan by 21–33% and 8–24%, respectively (Table 1).

The amino acid content in the yeasts was reduced after autolysis (by 2.4 and 5.9% in CJ and WA, respectively), which is also reflected by the increase in non-protein nitrogen content of the yeasts by 13 and 10% in CJ and WA, respectively (Table S1).

### 2.2. Fish Performance and Nutrient Digestibility

There was no significant difference (*p* > 0.05) in biomass gain, specific growth rate (SGR), feed intake and feed conversion ratio (FCR) among the dietary treatments (Table S2). During the first week of the experiment, feed intake was low for all dietary groups (Figure S1). No fish mortality was observed throughout the experimental period. The apparent digestibility coefficient (ADC) of crude protein was significantly (*p* < 0.05) higher in fish fed the FM diet compared with the other dietary treatments (Table S2). Conversely, fish fed the IWA diet had significantly lower (*p* < 0.05) digestibility of crude lipids compared with the other treatments (Table S2).

### 2.3. Histopathological Changes in Fish

**Figure 1:**
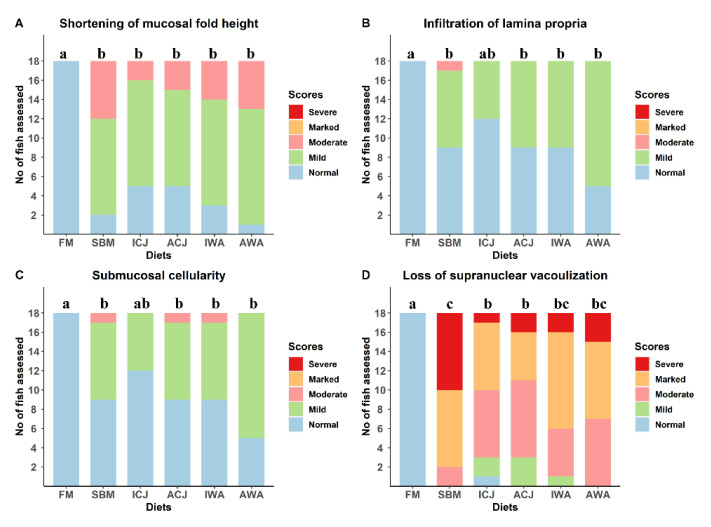
Histopathological changes in the distal intestine of Atlantic salmon smolts fed FM- or SBM-based diets with yeasts in seawater. The semi-quantitative scoring was obtained by measuring changes in four morphological parameters: (**A**) shortening of mucosal fold height; (**B**) infiltration of lamina propria; (**C**) submucosal cellularity; and (**D**) loss of supranuclear vacuolization. Each parameter was given a score of “0” representing normal morphology; “1” mild; “2” moderate; “3” marked; and “4” severe enteritis. Groups with different letters (a–c) above the bar charts are significantly different (*p* < 0.05). The diets were: FM—fishmeal-based; SBM—soybean meal-based; 4 experimental diets containing 300 g/kg SBM and 100 g/kg of ICJ—inactivated *Cyberlindnera jadinii*; ACJ—autolyzed *C. jadinii*; IWA—inactivated *Wickerhamomyces anomalus*; AWA—autolyzed *W. anomalus* diets.

Mild to moderate inflammatory changes were observed in the DI mucosa of fish fed the experimental diets (Figure S2). The observed changes were characterized by a marked to total loss in enterocyte vacuolization, a mild to moderate decrease in mucosal fold height, and a mild infiltration of the submucosa and lamina propria by inflammatory cells (Figure S2). Fish fed the FM diet showed normal and healthy morphology (Figure 1a–d). Mild to moderate inflammatory changes were observed in fish fed the SBM diet and were not statistically different (*p* > 0.05) from fish fed ICJ, ACJ, IWA, and AWA diets (Figure 1a–c). Considering histological changes due to loss of supranuclear vacuolization, fish fed ICJ and ACJ diets were significantly different (*p* < 0.05) from fish fed SBM with marked changes (Figure 1d).

### 2.4. Changes in T-Lymphocyte Population

**Figure 2:**
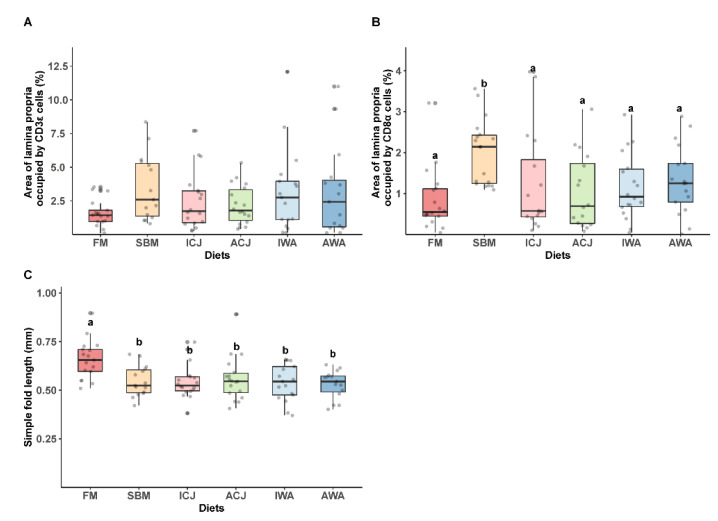
Area of lamina propria occupied by (**A**) CD3ε and (**B**) CD8α T-cells, and (**C**) simple fold length of the distal intestine of Atlantic salmon smolts fed FM- or SBM-based diets with yeasts in seawater. Groups with different letters (a–b) above the boxplots are significantly different (*p* < 0.05). The diets were: FM—fishmeal-based; SBM—soybean meal-based; 4 experimental diets containing 300 g/kg SBM and 100 g/kg of ICJ—inactivated *Cyberlindnera jadinii*; ACJ—autolyzed *C. jadinii*; IWA—inactivated *Wickerhamomyces anomalus*; AWA—autolyzed *W. anomalus* diets.

Both CD3ε- and CD8α-labeled cells were observed in all dietary groups with their expression more pronounced in the epithelium than the lamina propria (Figure S3). In general, there was a higher abundance of CD3ε positive lymphocytes compared with CD8α positive lymphocytes in the DI of fish fed all the experimental diets, (Figure S3). No statistical difference was observed for the area of epithelium occupied by CD3ε and CD8α positive cells among the diets (data not shown). Similarly, there was no significant difference (*p* > 0.05) for the area of lamina propria occupied by CD3ε positive cells among the diets (Figure 2). The area of lamina propria occupied by CD8α positive cells was significantly higher (*p* < 0.05) in fish fed the SBM diet, compared with the other diets (Figure 2). The simple fold length was significantly higher in fish fed the FM diet, compared with fish fed SBM, ICJ, ACJ, IWA and AWA diets (Figure 2).

### 2.5. Transcriptomics

Higher differentially expressed genes (DEGs) were found between diet comparisons in DI tissue compared with spleen tissue (Table 2). In the DI, the comparison between fish fed SBM and FM diets showed 173 down-regulated and 143 up-regulated genes. A lower number of DEGs occurred when fish fed ICJ (71 down-regulated, 54 up-regulated) and ACJ (33 down-regulated, 31 up-regulated) diets were compared with fish fed the FM diet. The highest number of DEGs were observed in fish fed AWA (2685 down-regulated, 2714 up-regulated) and IWA (1299 down-regulated, 1036 up-regulated) diets, compared with those fed the FM diet. A list of significant DEGs between diet comparisons along with the name of each gene is attached in Table S3.

### 2.6. Gene Ontology

**Figure 3:**
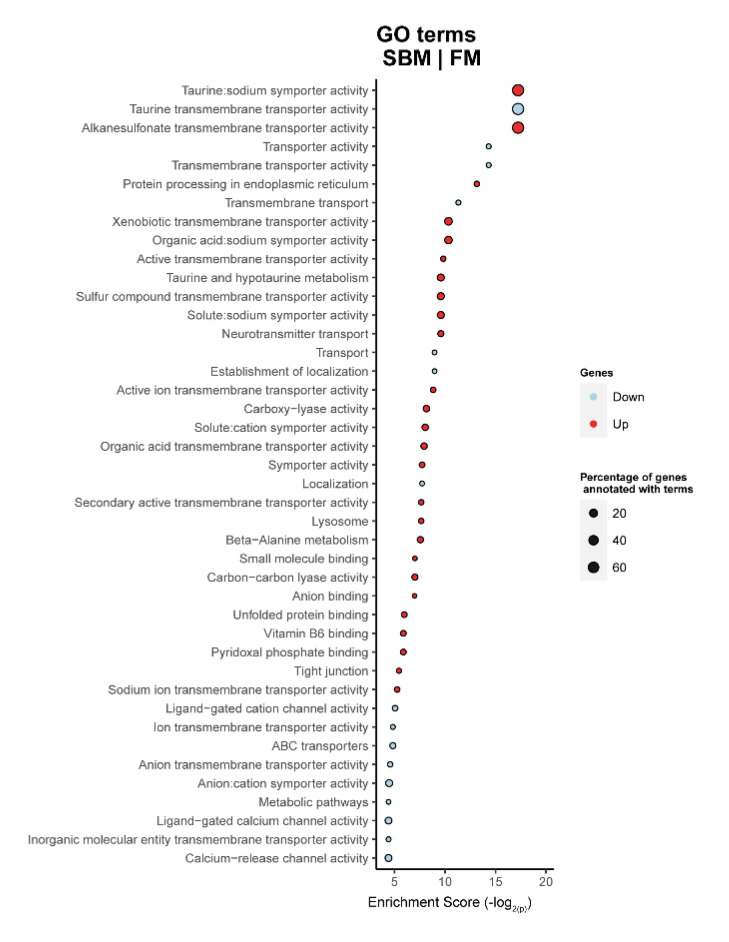
Significantly enriched gene ontology (GO) terms (minGSSize = 3) in the distal intestine of Atlantic salmon smolts fed SBM-based diet compared with fish fed the FM-based diet. The list is ordered by decreasing Enrichment Score (−log_2_(P)). Up, up-regulated (in red); Down, down-regulated (in light blue). The diets were: FM—fishmeal-based and SBM—soybean meal-based diets.

For DI tissue, the Gene Ontology (GO) analysis when comparing fish fed SBM with those fed the FM diet, showed that the up-regulated terms in SBM were mainly related to transport-channel activity, lysosome, and tight junction function, while the down-regulated GO terms were related to metabolic pathways (SBM|FM, Figure 3).

**Figure 4:**
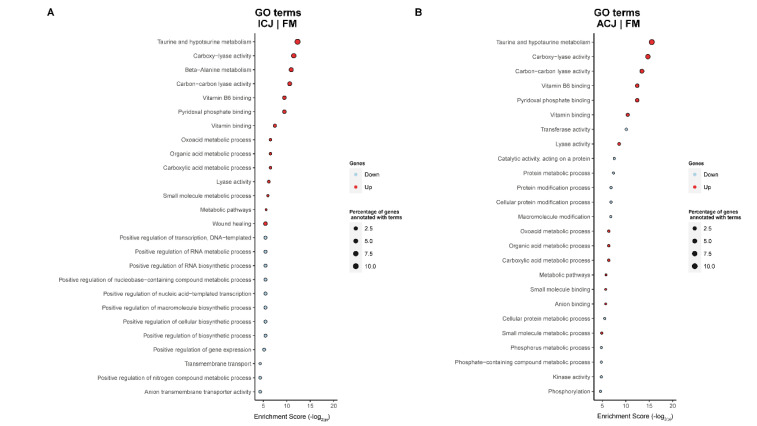
Significantly enriched gene ontology (GO) terms (minGSSize = 3) in the distal intestine of Atlantic salmon smolts fed ICJ (**A**) or ACJ (**B**) diets compared with fish fed the FM diet. The list is ordered by decreasing Enrichment Score (-log_2_(P)). Up, up-regulated (in red); Down, down-regulated (in light blue). The diets were: FM—fishmeal-based; and diet containing 300 g/kg SBM with 100 g/kg of ICJ—inactivated *Cyberlindnera jadinii* and ACJ—autolyzed *C. jadinii* diets.

The comparison between fish fed ICJ and FM revealed up-regulated GO terms relating to metabolic process, wound healing, vitamin B6 binding, taurine and hypotaurine metabolism, while the down-regulated GO terms were related to transport activity and biosynthetic processes (ICJ|FM, Figure 4A). Similar up-regulated and down-regulated GO terms were observed when fish fed ACJ were compared with those fed FM (ACJ|FM, Figure 4B). 

**Figure 5:**
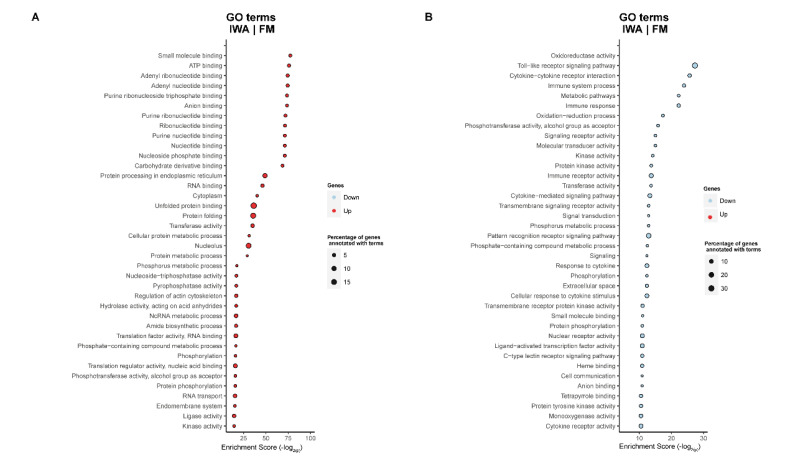
Significantly enriched gene ontology (GO) terms (minGSSize = 3) in the distal intestine of Atlantic salmon smolts fed the IWA diet compared with fish fed the FM diet. The list is ordered by decreasing Enrichment Score (−log_2_(P)). (**A**). Up, up-regulated (in red); (**B**). Down, down-regulated (in light blue). The diets were: FM—fishmeal-based; and diets containing 300 g/kg SBM with 100 g/kg of IWA—inactivated *Wickerhamomyces anomalus* diet. The top 40 up-regulated and down-regulated genes are presented.

In addition, when comparing fish fed IWA and AWA diets with those fed the FM diet, the results showed up-regulated terms related to energy metabolism (Figure 5A and Figure S4A), while the down-regulated GO terms were related to the immune response pathway and oxidation–reduction process (Figure 5B and Figure S4B). There was no differentially significant GO term between diet comparisons for the spleen tissue.

**Figure 6:**
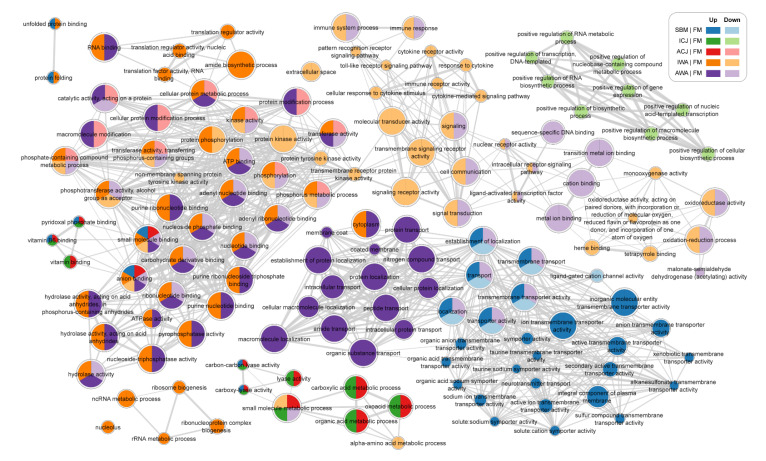
Network of significantly enriched GO terms between different diet comparisons. Each node is a GO term with size indicating the number of genes annotated with that term. Edges connect GO terms that are sufficiently similar to each other in terms of shared genes with edge thickness indicating the similarity coefficient (>0.375). Node colors indicate diet comparisons in which the GO term was significantly enriched, according to the color legend. The diets were: FM—fishmeal-based; SBM—soybean meal-based; 4 experimental diets containing 300 g/kg SBM and 100 g/kg of ICJ—inactivated *Cyberlindnera jadinii*; ACJ—autolyzed *C. jadinii*; IWA—inactivated *Wickerhamomyces anomalus*; AWA—autolyzed *W. anomalus diets*. Cytoscape was used to visualize enriched GO terms for all diet comparisons in a network.

Figure 6 showed shared pathways of network analysis of GO terms when comparing fish fed SBM-based diets with those fed the FM diet. Fish fed SBM and AWA diets shared similar pathways associated with transporter activity. The shared pathways were up-regulated in SBM and down-regulated in AWA. Fish fed ICJ and ACJ diets shared similarities associated with up-regulation of the acid metabolic process, lyase activity and small molecule metabolic process. Furthermore, we observed that fish fed IWA contained down-regulation of pathways related to the toll-like receptor signaling pathway, C-lectin receptor pathway, cytokine receptor activity and signal transduction, and these are connected to other pathways that were down-regulated in fish fed the AWA diet. Furthermore, many pathways related to nucleotide and carbohydrate binding and hydrolase, phosphatase, and ATPase are up-regulated in fish fed both IWA and AWA diets. 

## 3. Discussion

The feed intake and growth data showed that the fish adjusted quickly to the seawater environment and obtained a reasonable feed intake two weeks post transfer. The length of the current study is too short to draw any conclusions on fish performance, but 10% dietary inclusion of the two yeast species did not compromise the growth of Atlantic salmon smolts. The reduced digestibility of crude protein in fish fed SBM-based diets reflected in numerically lower performance in fish fed these diets. In line with previous studies [4,5], fish fed the SBM diet developed classical symptoms of SBMIE, characterized by marked to severe loss in enterocyte vacuolization. The current results showed that the inclusion of ICJ and ACJ yeasts partially reduced the loss of supranuclear vacuolization in Atlantic salmon fed SBM-based diets. This is in accordance with previous studies, which demonstrated that CJ can be used to alleviate SBMIE in Atlantic salmon [15,16,17]. Based on the histological results, the inclusion of IWA and AWA yeasts in the diets did not prevent SBMIE, which contradicts the results of a recent work [15]. The WA used in the previous study [15], however, had a higher content of crude protein (19% more), mannan (38% more) and chitin (75% more) compared with the currently used batch. Conversely, the β-glucan content of the previous WA yeast was 31% lower than the present one. The disparity in the composition of bioactive components present in the yeast batch could account for their variation in preventing SBMIE in Atlantic salmon. The limited effect of WA on SBMIE in the present study could also be attributed to the difference in fish production stage, fish size or age, or to the severity of SBMIE. The previous study by Agboola et al. [15] was conducted in freshwater with 5 g fish, which only displayed mild inflammatory changes when fed the SBM diet [15], suggesting that WA might be effective against mild inflammatory changes in Atlantic salmon.

Previous studies have demonstrated that T-cell mediated hypersensitivity is crucial to the development of SBMIE in Atlantic salmon [19,20,21]. Thus, CD3ε and CD8α positive cells are important biomarkers to evaluate inflammation caused by inclusion of SBM (or other plant ingredients) in salmon diets [17,19]. Furthermore, they are important biomarkers for studying the effect of functional diets on inflammatory responses of fish fed SBM [17]. In the present study, the higher abundance of both T-lymphocyte populations along the basal part of DI epithelium, compared with the lamina propria, was in accordance with a previous study [17]. Bakke-McKellep et al. [19] reported an increased presence of CD3ε-labeled cells in the lamina propria of fish presenting SBMIE, which is contrary to the observation in the present study. The discrepancy might be attributed to a number of factors such as variation in SBM used and increased tolerance of fish to SBM in recent years. Previous studies have shown that differences in the level of ANFs (especially saponin) in commercial sources of SBM can influence the degree of SBMIE in Atlantic salmon [22,23,24]. The increased tolerance of fish in recent years could be the result of breeding and genetic selection of fish for improved growth performance and adaptability to plant-based diets. Studies have shown increased growth performance and no detection of enteritis in strain of rainbow trout selected on a diet containing SBM, compared to non-selected strains [25,26]. However, similar studies in Atlantic salmon are scarce in scientific literature. 

The area of lamina propria occupied by CD8α positive cells were higher in fish fed the SBM diet compared with those fed the FM diet, which is in line with previous studies [17,19]. However, there was a reduction in the population of CD8α cells in fish fed ICJ, ACJ, IWA and AWA diets compared with fish fed SBM, indicating an immunomodulatory effect of these yeasts when included in SBM-based diets. Reveco-Urzua et al. [17] reported a similar effect when 2.5% CJ was supplemented to a 20% SBM-based diet. Our results showed there was no effect of yeast supplementation on CD3ε cell population, and this could be explained by the large variability within the dietary group. 

Our results at the transcriptional level revealed that fish fed SBM compared with FM, showed activation of transporters and channel activities, implying increased permeability in the DI of fish. In the current study, increased gut permeability is supported by up-regulation of solute carrier proteins, slc6a6 and SC6A6, in the DI of fish fed the SBM diet. This is similar to findings of previous studies that showed that SBM increases gut permeability through the up-regulation of genes associated with solute carriers and channel proteins [6,8,27]. The results also showed that fish fed SBM responded to inflammatory changes through up-regulation of genes associated with tight junction and lysosomal pathways. Tight junction proteins play important roles in intestinal fluid permeability in Atlantic salmon [28,29,30,31]. In this study, tight junction proteins such as aquaporin (aqp10b), claudin (cldn12) and nucleoporin (nup93) genes were up-regulated in response to feeding SBM. These have been previously reported in the intestine of Atlantic salmon fed SBM diets in response to increased gut permeability [8,28,32,33]. Lysosome is a vesicle that contains lysozyme, an anti-microbial protein responsible for pathogen degradation during innate immunity [34]. In this study, the up-regulation of the lysosomal pathway in fish fed SBM could be attributed to the translocation of opportunistic bacteria to the underlying mucosa due to increased intestinal permeability [9,10]. This result is in accordance with previous findings showing increased lysozyme production in the DI of Atlantic salmon fed SBM-based diet [6,35]. 

When comparing fish fed both CJ diets with FM, the results showed up-regulation of GO terms such as wound healing, as well as taurine and hypotaurine metabolism. Thus, it seems that the ICJ and ACJ yeasts were able to partially restore the integrity of the intestinal surface barrier following inflammatory damage caused by SBM. In response to the intestinal damage, genes participating in the wound healing process, such as vimentin (VIME) and integrin protein (itgb3a), were activated in fish fed ICJ and ACJ diets. Vimentin (VIME) is known to interact with other structural proteins such as microtubules to maintain cellular integrity and provide resistance to cell damage [36]. Integrin protein (itgb3a) mediates the adhesive properties of intestinal epithelial cells and is needed to achieve mucosal wound closure [37]. Integrin interacts with the actin-binding protein, annexin A2, to facilitate movement of intestinal epithelial cells during wound resealing [37]. Our speculation is that the bioactive compounds in CJ support the wound resealing process, and this is partially responsible for its mode of action in counteracting SBMIE. Another possible mode of action of ICJ and ACJ may be connected to the reduction in oxidative stress through taurine and hypotaurine pathways. Taurine is categorized as a semi-essential keto acid that plays a key role in innate immunity and reduction in oxidative stress [38,39]. 

Fish fed IWA and AWA diets revealed alteration of genes associated with the immune responses compared with those fed the FM diet. Cytokines, such as tumor necrosis factor alpha (TNFα), interleukin-12 (IL-12), interferon gamma (IFNγ) and pattern recognition receptor (e.g., Toll-like receptor-7 (TLR7)) were down-regulated in fish fed IWA and AWA diets. This implies that these yeasts were able to control the inflammatory profile of SBM associated with M1 macrophages [40]. We speculate that the down-regulation of IL-12 reduced the inflammatory profile associated with SBM by suppressing the expression of TNFα and IFNγ. Previous studies have demonstrated increased expression TNFα and IFNγ in Atlantic salmon in response to dietary SBM [7,15]. Thus, the down-regulation of TNFα and IFNγ genes in the DI of fish fed IWA and AWA diets indicates the potential of these yeasts to regulate the inflammation caused by SBM. The suppression of genes associated with TNFα and IFNγ in fish fed IWA and AWA diets is supported by the down-regulation of genes associated with CD83 expression. In higher vertebrates, CD83 is expressed by mature dendritic cells, and its down-regulation can suppress T helper cells and, in the process, decrease the expression of TNFα and IFNγ cytokines [41,42]. Although the possible immunomodulatory potential of IWA and AWA yeasts was detected on a transcriptomic level, this effect was not clearly seen on the histological level. 

Furthermore, the similar response of enriched pathways in fish fed the inactivated or autolyzed yeast diets (i.e., between ICJ vs. ACJ and IWA vs. AWA) suggests that processing by autolysis did not improve the beneficial health effect of the two yeast species in the current study, which is in contradiction with previous findings [7,15]. It is not clear what causes the discrepancy, but it might be related to the time lag between harvesting and autolysis of the yeast pastes in these experiments. The yeast pastes used by Agboola et al. [15] were stored for 5–6 months before the autolysis process. During this storage period, the yeasts might have undergone self-hydrolysis, which possibly contributes to their immunomodulatory effect in fish. On the contrary, yeasts used in the current study were autolyzed immediately after harvesting.

In the current study, the transcriptomic analysis of the spleen tissue revealed that the experimental diets did not induce systemic effects in fish. Previous studies on Atlantic salmon fed SBM-based diets have reported both similar effects [6] and contradicting effects [7] using head kidney and spleen, respectively. The reason for these differences is not clear, but it might be related to the sensitivity of immunological organs in the different life stages of fish. Similar to the current trial, Kiron et al.’s study [6] was conducted in seawater, whereas Morales-Lange et al.’s study [7] was conducted in freshwater. Thus, studies identifying possible factors influencing the systemic effect of fish fed SBM and SBM in combination with functional ingredients in both freshwater and seawater is warranted in the future. This might be key to our understanding of when to include functional ingredients in fish diets.

## 4. Materials and Methods

### 4.1. Yeast Production and Processing

In the present study, CJ and WA yeasts were cultured according to Lapeña et al. [43], using a growth medium containing a blend of enzymatic hydrolysates of pre-treated spruce wood (Picea abies) and chicken by-products. The yeast biomass was produced aerobically in a 42 L Techfors S bioreactor (Infors, Bottmingen, Switzerland) with 25 L working volume running as repeated batch fermentations. After harvesting, the yeasts were washed and re-suspended in 7 °C deionized water in a 30 L reactor, equipped with a helical impeller (Einar, Belach Bioteknik, Skogås, Sweden). The washed yeasts were further centrifuged to obtain a paste with 32% and 41% dry matter content for CJ and WA, respectively. The processing of the yeast paste was performed according to the method described by Agboola et al. [15]. Briefly, the paste from each yeast biomass was mixed and divided into two homogenous parts. The first part of the yeast paste was inactivated by spray-drying using a SPX 150 MS (SPX Flow Technology, Søborg, Denmark). The other part of the paste was autolyzed at 50 °C for 16 h in the 30 L Einar reactor, followed by spray-drying. The inlet and outlet temperatures of the spray-dryer were set at 180 and 80 °C, respectively. Inactivated CJ (ICJ), autolyzed CJ (ACJ), inactivated WA (IWA), and autolyzed WA (AWA) were the four yeast ingredients used in this study.

### 4.2. Formulation and Production of Fish Feeds

A total of six experimental diets were used in the current trial. The diets were a fishmeal-based (FM) control diet, a 30% soybean meal-based (SBM) diet as a challenging positive control diet, and four experimental diets containing 30% SBM and 10% inclusion of the different processed yeasts (ICJ, ACJ, IWA and AWA), respectively. The formulation and analyzed nutritional compositions of the experimental diets are presented in Table 3. The diets contained a similar ratio of digestible protein to digestible energy and were formulated to meet the nutritional requirement of Atlantic salmon smolts [44,45]. The plant-based diets were supplemented with crystalline lysine and methionine to balance the essential amino acid profile to that of the FM control. For feed production, all dry ingredients were weighed and mixed in a Spiry 25 dough mixer (Moretti Forni, Mondolfo, Italy). Gelatin was firstly mixed in cold water, then heated up to 60 °C in a microwave oven. The gelatin and fish oil were mixed with dry ingredients using the same mixer as described above. The mash was cold-pelleted using a P35A pasta extruder (Italgi, Carasco, Italy) and the resulting feed pellets were dried (to about 93% dry matter content) in small experimental dryers at about 60 °C drying temperature. The feeds were stored at 4 °C before and during the experimental period.

### 4.3. Fish Management and Feeding

A total of 450 vaccinated Atlantic salmon smolts with an average initial weight of 136 ± 0.25 g were sorted, batch weighed and transported in oxygenated plastic bags from the Center for sustainable Aquaculture at the Norwegian University of Life Sciences (Ås, Norway) to the research facility of Norwegian Institute of Water Resources (NIVA, Solbergstrand, Norway). The fish were randomly distributed into 18 fiber tanks (300 L) equipped with automatic feeders with 25 fish stocked into each tank. The six experimental diets were randomly allocated to all the tanks in triplicate. During the first week of the experiment, fish were fed 1% of their body weight, and feeding was subsequently increased based on feed consumption in each tank. Feeds were supplied 6 h a day using automatic feeders delivering feed every 12 min. Uneaten feed was collected after each feeding from the outlet water settling on a screen for each tank. Daily feed intake was calculated from the dry weight of the feed given and the dry weight of the recovered uneaten feed, corrected for feed recovery rate of each tank. Water salinity was gradually increased from 5 ppt at the start, until it reached full salinity (33 ppt) over the first 12 days of the experiment. Fish were kept under a 24 h light regime in a flow-through system with an average water temperature of 11.5 °C and average oxygen saturation of 84%. The water flow was kept at an average of 5.5 L min^−1^ during the experimental period. 

After 42 days of feeding, six fish were randomly selected from each tank, anesthetized with metacaine (MS-222, 50 mg L^−1^ water), and killed with a sharp blow to the head for tissue sampling. The body weight of individual selected fish was recorded and included in the total tank mean weight. After dissection, distal intestine (DI) and spleen tissue samples were collected from each selected fish. The DI is described as the segment from the increase in intestinal diameter and the appearance of transverse lumina folds to the anus. The DI was dissected longitudinally, the content was removed, and the tissue was carefully divided into two parts. One part was fixed in 10% phosphate-buffered formalin for 24 h before storage in 70% ethanol until further processing for both histological and immunohistochemistry analyses. The second part of the DI was cut into three pieces and immediately suspended in RNA-later and stored over night at 4 °C, and later at −80 °C until total RNA extraction. The spleen samples were treated in the same manner as the DI samples for total RNA extraction. The remaining fish per tank after tissue sampling were anesthetized, counted and group weighed for determination of fish growth performance. Furthermore, fecal samples were collected by stripping the remaining fish per tank for determination of nutrient digestibility. The fecal samples were stored at −20 °C before freeze-drying.

### 4.4. Histological Examination

The DI tissue samples (18 samples per dietary group) were processed at the Veterinary Institute Laboratory in Oslo, Norway, according to standard techniques for histological assessment [4]. Briefly, formalin-fixed DI tissue samples were dehydrated in ethanol, equilibrated in xylene, and embedded in paraffin. Longitudinal sections of 3 µm thickness from each DI tissue sample were prepared and stained with hematoxylin and eosin. The sections were then blindly characterized under a light microscope with an emphasis on the morphological changes observed with SBMIE as previously described by Baeverfjord and Krogdahl [4]. The histological scores were obtained through a semi-quantitative scoring system, measuring changes in four morphological criteria: shortening of mucosal fold height, increase in width and cellularity of the submucosa and lamina propria, and loss of enterocyte supranuclear vacuolization [4]. Each criterion was given a score of 0–4, where 0 represented normal; 1 mild changes; 2 moderate changes; 3 marked changes; and 4 severe changes. 

### 4.5. Detection of T-Lymphocytes by Immunohistochemistry

The CD3ε and CD8α positive T-lymphocytes in the DI tissue of fish fed the experimental diets were detected using immunohistochemistry, following previously described protocol [17]. Briefly, for labeling of CD3ε and CD8α positive T-lymphocytes, primary monoclonal antibody (CD3ε mouse anti-trout) at 1:600 [46] and primary monoclonal antibody (CD8α mouse anti-salmon) at 1:50 [47] were used. The slides were incubated at room temperature for 1 h, followed by 30 min incubation with secondary antibody. Sections labeled for CD3ε were incubated with secondary antibody kit polymer-HRP anti-mouse (DAKO En Vision+ System-HRP, Dako, Glostrup, Denmark) while sections labeled for CD8α were incubated with biotinylated secondary anti-mouse IgG antibody (BA-9200, Vector laboratories, Burlingame, CA, USA). Peroxidase activity in the CD3ε and CD8α slides were detected with 3,3′ diaminobenzidine (DAKO En Vision+ System-HRP, Dako, Glostrup, Denmark) and 3-amino-9-ethylcarbazole (SK-4205, Vector laboratories, Burlingame, CA, USA); respectively. Sections for both T-lymphocytes were counter-stained with hematoxylin for 10s and mounted using an Aquatex (Merck, Darmstadt, Germany). Slides incubated without the primary antibodies were included as negative controls for both T-lymphocytes. To estimate the area of lamina propria occupied by CD3ε and CD8α-labeled cells, QuPath digital pathology software (v0.2.3) [48] was used with some modifications to the previously described method of Reveco-Urzua et al. [17]. Fold length was measured from the stratum compactum to the tip of the simple fold using ImageJ software (v1.53c).

### 4.6. RNA Isolation, Library Preparation and RNA Sequencing

Total RNA was extracted from DI and spleen tissues collected from 36 fish (6 fish per dietary group) using Qiazol Lysis Reagent (Qiagen, Hilden, Germany) and chloroform following the protocol described by Toni et al. [49]. Thereafter, the RNA concentration was quantified using a NanoDrop TM 8000 spectrophotometer (Nanodrop Technologies, Wilmington, DE, USA). RNA integrity was determined using a 2100 Bioanalyzer (Agilent Technologies, Santa Clara, CA, USA). All samples showed high-quality integrity (RIN ≥ 8). Library preparation using TruSeq Stranded mRNA library prep (Illumina, San Diego, CA, USA) was performed at the Centre for Integrative Genetics (CIGENE, NMBU, Ås, Norway). Libraries were pooled and RNA sequencing was performed using the Illumina NovaSeq S4 platform (150 bp paired-end reads) at the Norwegian Sequencing Center (UiO, Oslo, Norway).

### 4.7. Data Analysis of RNA Sequencing

RNA sequence data analysis was performed using the publicly available nf-core/RNA-seq pipeline version 3.3 implemented in Nextflow 21.04.0 [50]. In brief, raw reads were trimmed using Trim Galore and clean reads were thereafter aligned to Salmo salar genome Ssal_v3.1 (GenBank assembly accession: GCA_905237065.2) by STAR_RSEM. Gene-level assignment was performed using featureCounts (v1.4.6). Differentially expressed genes (DEGs) between diets were estimated using DESeqe2 (v1.22.1) and SARTools (v1.7.3) R packages. All genes with a log_2_ fold change > 2 or < −2 were designated up or down, respectively. Significant DEGs were determined when the adjusted *p*-value was < 0.05. To characterize DEGs, gene ontology (GO) enrichment using three categories (molecular function, biological process, and cellular components) was performed with the ShinyGO (v0.741) online tool, applying the False Discovery Rate (FDR) correction for multiple testing [51]. GO categories were selected (minGSSize = 3) and displayed as Enrichment Score (–log_2_(P). EnrichmentMap v3.3.3 [52] in Cytoscape v3.8.1 [53] was used to visualize enriched GO terms for all diet comparisons in a network. Two GO terms were connected if their similarity coefficient (mean of overlap and Jaccard coefficients) was greater than 0.375.

### 4.8. Composition of Yeasts and Fish Feeds

The nutritional compositions of yeasts and feeds were determined according to a previously described protocol by Agboola et al. [54]. The composition of yeast cell wall was estimated without prior cell wall isolation following the method described by Hansen et al. [55]. Briefly, the yeasts were hydrolyzed with sulphuric acid and the liberated sugar monomers (mannose, N-acetylglucosamine and glucose) were quantified by high-performance anion-exchange chromatography with pulsed amperometric detection [55], to determine the mannan, chitin and glucan content of the yeast cell wall.

### 4.9. Calculations and Statistical Analyses

The biomass gain, SGR and FCR were calculated based on the equations expressed in Agboola et al. [54]. In brief, the biomass gain was expressed as the difference between the average final weight and initial body weight of fish per tank. The FCR was expressed as the ratio between average feed intake per day and average biomass gain per day. The SGR was calculated as the logarithm differences between the average final and initial weight of fish divided by the experimental period. The ADCs of nutrients in the diets were determined based on the equation of Cho and Slinger [56]. 

Fish performance, nutrient digestibility, and area of lamina propria covered with T-lymphocytes were tested for treatment effects using one-way ANOVA. Significance difference (*p* < 0.05) among diets were detected using Tukey’s HSD test. These analyses were performed using the SPSS statistical software package version 27 (IBM Institute, Armonk, NY, USA). Differences in histological scores for the various evaluated morphological characteristics of the DI tissue were analyzed for statistical significance using ordinal logistic regression in the R statistical package (version 3.6.2; 2019). Differences were examined based on odds ratios of the different feeding groups having different histology scores compared to the FM diet.

## 5. Conclusions

The results demonstrate that the inclusion of CJ yeast reduced the loss of supranuclear vacuolization and decreased population of CD8α positive cells in the DI of fish fed SBM-based diets. Inclusion of CJ and WA yeasts also induced transcriptomic changes related to wound healing and immune response pathways in fish fed SBM-based diets. Processing by autolysis did not improve the beneficial health effect of CJ and WA yeasts. This study suggests that the yeast species *Cyberlindnera jadinii* and *Wickerhamomyces anomalus* are novel high-quality protein sources with health-beneficial effects in terms of reducing inflammation associated with feeding plant-based diets to Atlantic salmon.

## Figures and Tables

**Table 1:** Composition of spray-dried yeasts with and without the autolysis treatment. All values are presented in % DM, except gross energy, which is presented in MJ/kg DM.

	*Cyberlindnera jadinii*		*Wickerhamomyces anomalus*	
	Inactivated	Autolyzed	Inactivated	Autolyzed
DM ^1^ (%)	96.3 ± 0.03	93.1 ± 0.04	96.1 ± 0.02	96.1 ± 0.06
**Nutrients (% DM) ^2^**				
Crude protein	46.5 ± 0.47	47.4 ± 0.01	43.0 ± 0.04	42.1 ± 0.26
Crude lipids	2.9 ± 0.18	5.7 ± 0.17	2.8 ± 0.06	4.1 ± 0.02
Ash	5.7 ± 0.00	5.9 ± 0.01	5.5 ± 0.00	5.5 ± 0.00
Total phosphorus	0.6 ± 0.02	0.6 ± 0.01	0.5 ± 0.01	0.4 ± 0.02
Gross energy (MJ/kg DM)	21.8 ± 0.01	22.32 ± 0.02	21.1 ± 0.01	21.5 ± 0.01
**Cell wall polysaccharides (% DM) ^3^**				
β-glucan	16.4 ± 3.19	11.1 ± 0.84	15.0 ± 1.41	11.8 ± 0.73
Mannan	7.9 ± 2.16	6.0 ± 0.66	11.3 ± 0.95	10.4 ± 0.67
Chitin	0.3 ± 0.07	0.2 ± 0.02	0.5 ± 0.05	0.4 ± 0.08

^1^ DM—dry matter; ^2^ Crude protein, crude lipids, ash, total phosphorus, and gross energy contents of yeasts are mean values ± SD from duplicate analyses; ^3^ β-glucan, mannan and chitin contents of yeasts are mean values ± SD from triplicate analyses.

**Table 2:** Significant differentially expressed genes (DEGs) per diet-comparison ^1^.

Diet Comparison	Down-Regulated	Up-Regulated
***Distal intestine***		
SBM|FM	173	143
ICJ|FM	71	54
ACJ|FM	33	31
IWA|FM	1299	1036
AWA|FM	2685	2714
***Spleen***		
SBM|FM	6	20
ICJ|FM	15	11
ACJ|FM	5	12
IWA|FM	7	6
AWA|FM	12	19

^1^ The diets were: FM—fishmeal-based; SBM—soybean meal-based; 4 other diets containing 300 g/kg SBM and 100 g/kg of ICJ—inactivated *Cyberlindnera jadinii*; ACJ—autolyzed *C. jadinii*; IWA—inactivated *Wickerhamomyces anomalus*; AWA—autolyzed *W. anomalus* diets.

**Table 3:** Formulation and nutritional composition of the experimental diets *.

	FM	SBM	ICJ	ACJ	IWA	AWA
***Diet Formulation* ^a^**						
Fish meal ^b^	433.4	261.4	208.4	208.4	208.4	208.4
Soybean meal ^c^	0	300	300	300	300	300
Yeast ^n^	0	0	100	100	100	100
Wheat gluten ^d^	170	136	111	111	111	111
Potato starch ^e^	120	90	68	68	68	68
Cellulose	80	0	0	0	0	0
Fish oil ^f^	130	130	130	130	130	130
Gelatin ^g^	60	60	60	60	60	60
Monocalcium phosphate ^h^	0	10	10	10	10	10
Premix ^i^	5	5	5	5	5	5
L-lysine ^j^	0	3	3	3	3	3
DL-Methionine ^k^	0	3	3	3	3	3
Choline chloride ^l^	1.5	1.5	1.5	1.5	1.5	1.5
Yttrium oxide ^m^	0.1	0.1	0.1	0.1	0.1	0.1
***Diet Composition (Analyzed Values)* ^o^**						
Dry matter (g/kg)	926	897	889	889	924	913
Crude protein	531	542	518	530	519	521
Starch	131	103	92	93	89	87
Ash	78	77	75	75	74	74
Carbon	509	510	503	518	513	511
Sulphur	6.0	6.3	6.2	6.0	6.1	6.0
Energy (MJ/kg DM)	23.3	23.1	23.3	23.3	23.1	23.1
DP:DE ^p^	23.1	23.3	22.8	22.8	22.5	22.5

^a^ Diet formulations are expressed in g/kg; ^b^ LT fishmeal, Norsildmel, Egersund, Norway; ^c^ Soybean meal, Denofa AS, Fredrikstad, Norway; ^d^ Wheat gluten, Amilina AB, Panevezys, Lithuania; ^e^ Lygel F 60, Lyckeby Culinar, Fjälkinge, Sweden; ^f^ NorSalmOil, Norsildmel, Egersund, Norway; ^g^ Rousselot 250 PS, Rousselot SAS, Courbevoie, France; ^h^ Monocalcium phosphate, Bolifor MCP-F, Yara, Oslo, Norway; ^i^ Premix fish, Norsk Mineralnæring AS, Hønefoss, Norway. Per kg feed; Retinol 3150.0 IU, Cholecalciferol 1890.0 IU, α-tocopherol SD 250 mg, Menadione 12.6 mg, Thiamin 18.9 mg, Riboflavin 31.5 mg, d-Ca-Pantothenate 37.8 mg, Niacin 94.5 mg, Biotin 0.315 mg, Cyanocobalamin 0.025 mg, Folic acid 6.3 mg, Pyridoxine 37.8 mg, Ascorbate monophosphate 157.5 g, Cu: CuSulfate 5H_2_O 6.3 mg, Zn: ZnSulfate 151.2 mg, Mn: Mn(II)Sulfate 18.9 mg, I: K-Iodide 3.78 mg, Ca 1.4 g; ^j^ L-Lysine CJ Biotech CO., Shenyang, China; ^k^ Rhodimet NP99, Adisseo ASA, Antony, France; ^l^ Choline chloride, 70% Vegetable, Indukern SA., Spain; ^m^ Y_2_O_3_. Metal Rare Earth Limited, Shenzhen, China; ^n^ ICJ—inactivated *Cyberlindnera jadinii*; ACJ—autolyzed *C. jadinii*; IWA—inactivated *Wickerhamomyces anomalus*; AWA—autolyzed *W. anomalus*; ^o^ Diet compositions are expressed in g/kg dry matter (DM) unless otherwise stated; ^p^ DP:DE = Digestible protein to digestible energy ratio. Calculated using internal digestibility values of various ingredients; * The diets were: FM—fishmeal-based; SBM—soybean meal-based; 4 other diets containing 300 g/kg SBM and 100 g/kg of ICJ, ACJ, IWA and AWA yeasts.

## Data Availability

The obtained raw sequencing data were deposited in the Gene Expression Omnibus database (GEO-NCBI: GSE193239). Other raw data generated in this study are available from the corresponding authors, upon reasonable request.

## Supplementary Materials

The following supporting information can be downloaded at: https://www.mdpi.com/article/10.3390/ijms23031675/s1.
