# Supplementary material for: Effects of Yeast Species and Processing on Intestinal Health and Transcriptomic Profiles of Atlantic Salmon (Salmo salar) Fed Soybean Meal-Based Diets in Seawater
Source: Int J Mol Sci. 2022 Jan 31;23(3):1675. doi: 10.3390/ijms23031675 (PMC8836103; doi:10.3390/ijms23031675)
Supplement: Supplementary file 1 [file ijms-23-01675-s001.zip › Supplementary file S1.pdf]

International Journal of
Molecular Sciences

MDPI

TABLE S1 Amino acid composition of yeasts with and without the autolysis treatment, after the drying process[1].

	Cyberlindnera jadinii		*Wickerhamomyces anomalus*	
	Inactivated	Autolyzed	Inactivated	Autolyzed
Essential amino acids (g/kg DM)[2]				
Arginine	25.5	20.4	22.6	18.9
Histidine	10.7	11.0	10.2	10.1
Isoleucine	14.6	15.5	13.4	13.5
Leucine	24.4	25.1	22.0	22.0
Lysine	23.9	24.9	24.3	23.6
Methionine	5.4	5.5	4.2	4.3
Phenylalanine	14.1	13.9	13.2	13.0
Threonine	17.1	17.5	15.1	15.1
Valine	14.8	15.8	12.7	12.8
Non-essential amino acids (g/kg DM)[2]				
Alanine	23.0	25.3	21.0	21.7
Aspartic acid	33.1	33.1	31.2	30.8
Glycine	24.3	25.5	21.9	22.0
Glutamic acid	62.3	50.5	62.6	48.7
Cysteine	3.4	3.3	2.8	2.6
Tyrosine	10.5	10.4	11.0	10.9
Proline	18.8	19.7	17.7	16.9
Serine	15.6	15.7	15.3	15.1
Sum amino acids[3]	341.3	333.2	321.3	302.2
Non-protein nitrogen[4]	124.2	140.5	108.3	119.1

[1]Values are presented as mean from duplicate analysis. The amino acid contents of yeasts are expressed using water corrected molecular weights.
[2]DM – dry matter.
[3]sum of amino acids = essential + non-essential amino acids.
[4]Non-protein nitrogen = crude protein – sum amino acids.

TABLE S2 Growth performance and apparent digestibility coefficient (%; ADC) of nutrients in experimental diets fed to Atlantic salmon in seawater.[1]

	FM	SBM	ICJ	ACJ	IWA	AWA	SEM[2]	*P-values*[3]
Growth performance								
Initial biomass (g/fish)	136.0	135.9	136.0	136.2	135.7	135.9	0.06	0.413
Final biomass (g/fish)	184.8	182.4	182.5	174.3	175.5	174.3	1.71	0.266
Biomass gain (g/fish/d)	1.16	1.11	1.11	0.91	0.95	0.91	0.04	0.306
Specific growth rate (%/d)	0.73	0.70	0.70	0.59	0.61	0.59	0.02	0.329
Feed intake (g/fish/d)	1.73	1.63	1.66	1.56	1.65	1.59	0.02	0.088
Feed conversion ratio	1.50	1.49	1.52	1.77	1.76	1.81	0.06	0.422
Apparent digestibility coefficient of nutrients (%; ADC)								
Dry matter	67.5^{a}	65.1^{ab}	65.0^{ab}	65.4^{ab}	62.6^{ab}	61.9^{b}	0.60	0.048
Crude protein	86.6^{a}	82.9^{b}	82.2^{b}	82.1^{b}	79.8^{b}	79.9^{b}	0.62	0.001
Crude lipids	92.6^{ab}	92.2^{abc}	94.2^{a}	92.5^{ab}	90.3^{c}	91.8^{bc}	0.33	0.003

[1]The diets were: FM – fishmeal-based; SBM – soybean meal-based; 4 other diets containing 300 g/kg SBM and 100 g/kg of ICJ – inactivated *Cyberlindnera jadinii*; ACJ – autolyzed *C. jadinii*; IWA – inactivated *Wickerhamomyces anomalus*; AWA – autolyzed *W. anomalus* diets.

[2]Standard error of mean.

[3]Means in the same row but with different superscript (a-c) denote significant ($P < 0.05$) difference among the experimental diets and was computed using Tukey comparison test. n is 3 replicate tanks per dietary group.

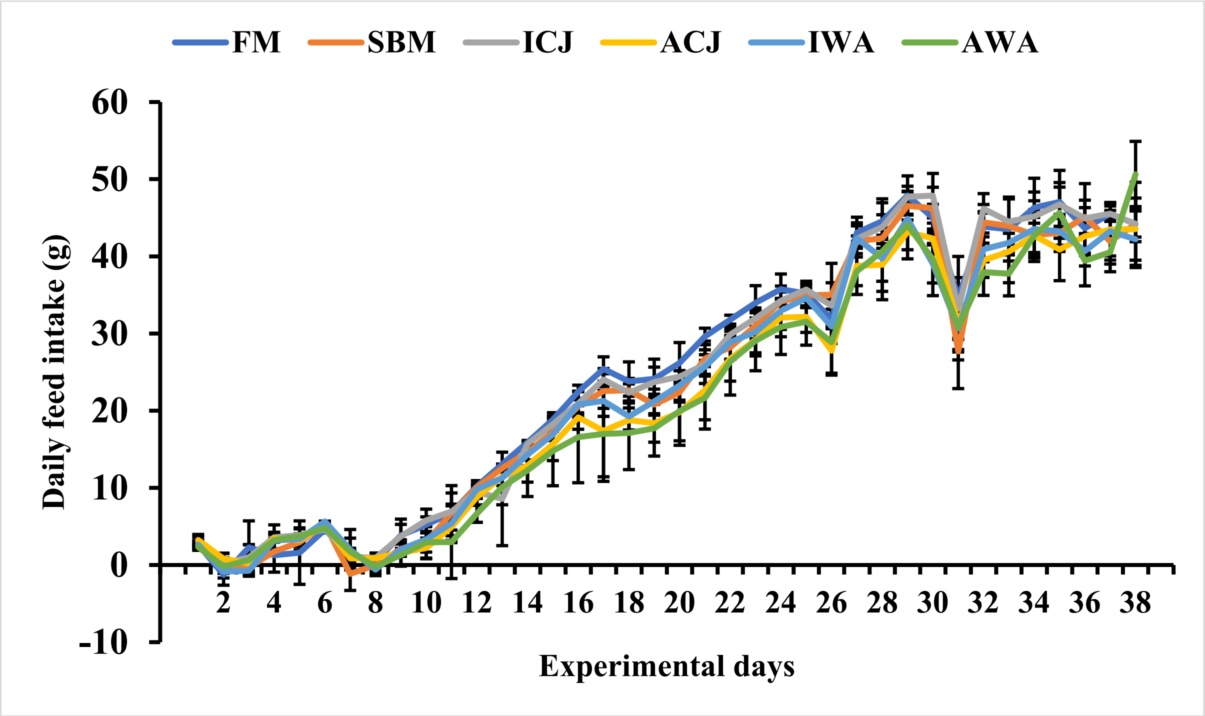

FIGURE S1 Average daily feed intake of Atlantic salmon fed the experimental diets in seawater. Each data point is the mean ± standard deviation of the three tanks per dietary group. FM – fishmeal-based; SBM – soybean meal-based; 4 other diets containing 300 g/kg SBM and 100 g/kg of ICJ – inactivated *Cyberlindnera jadinii*; ACJ – autolyzed *C. jadinii*; IWA – inactivated *Wickerhamomyces anomalus*; AWA – autolyzed *W. anomalus* diets.

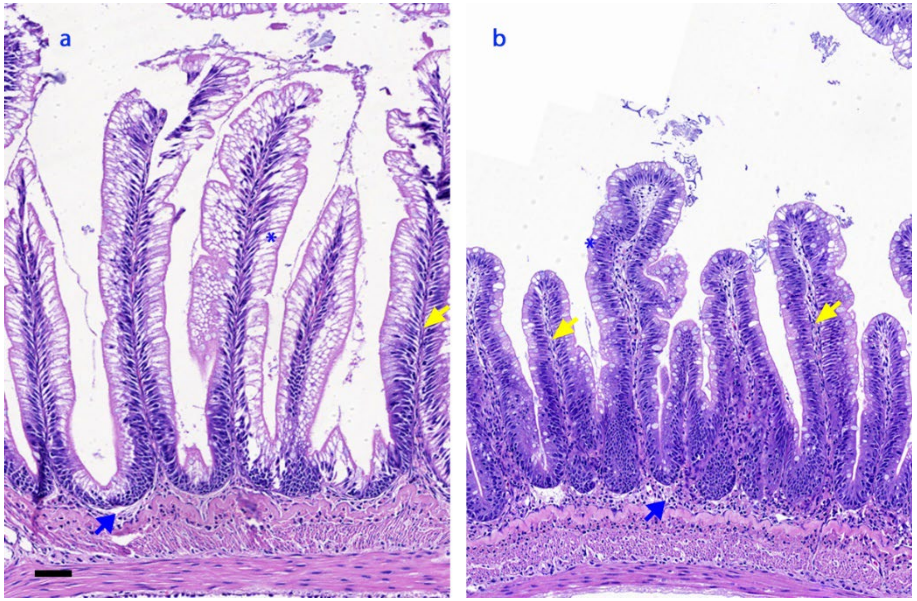

FIGURE S2 Representative images of distal intestinal sections showing **a**) normal and healthy gut and (**b**) gut with mild inflammation. Image **b** is characterized by infiltration of the submucosa (blue arrow) and lamina propria (yellow arrow); mild shortening of mucosal folds and loss of enterocyte supranuclear vacuolization (blue asterisks). Scale bar represents a distance of 50 µm.

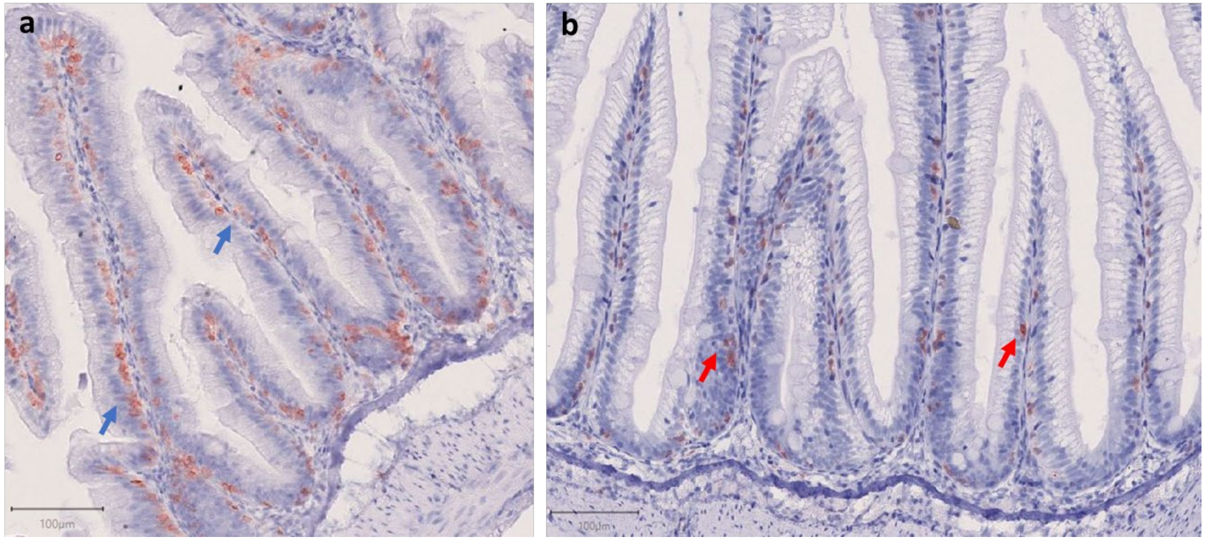

FIGURE S3 Representative images of distal intestine showing abundance of (**a**) CD3ε positive lymphocytes (blue arrow) compared with (**b**) CD8α positive lymphocytes (red arrow) in fish fed the experimental diets. Images are representatives of distal intestine of fish fed the soy-bean meal diet.

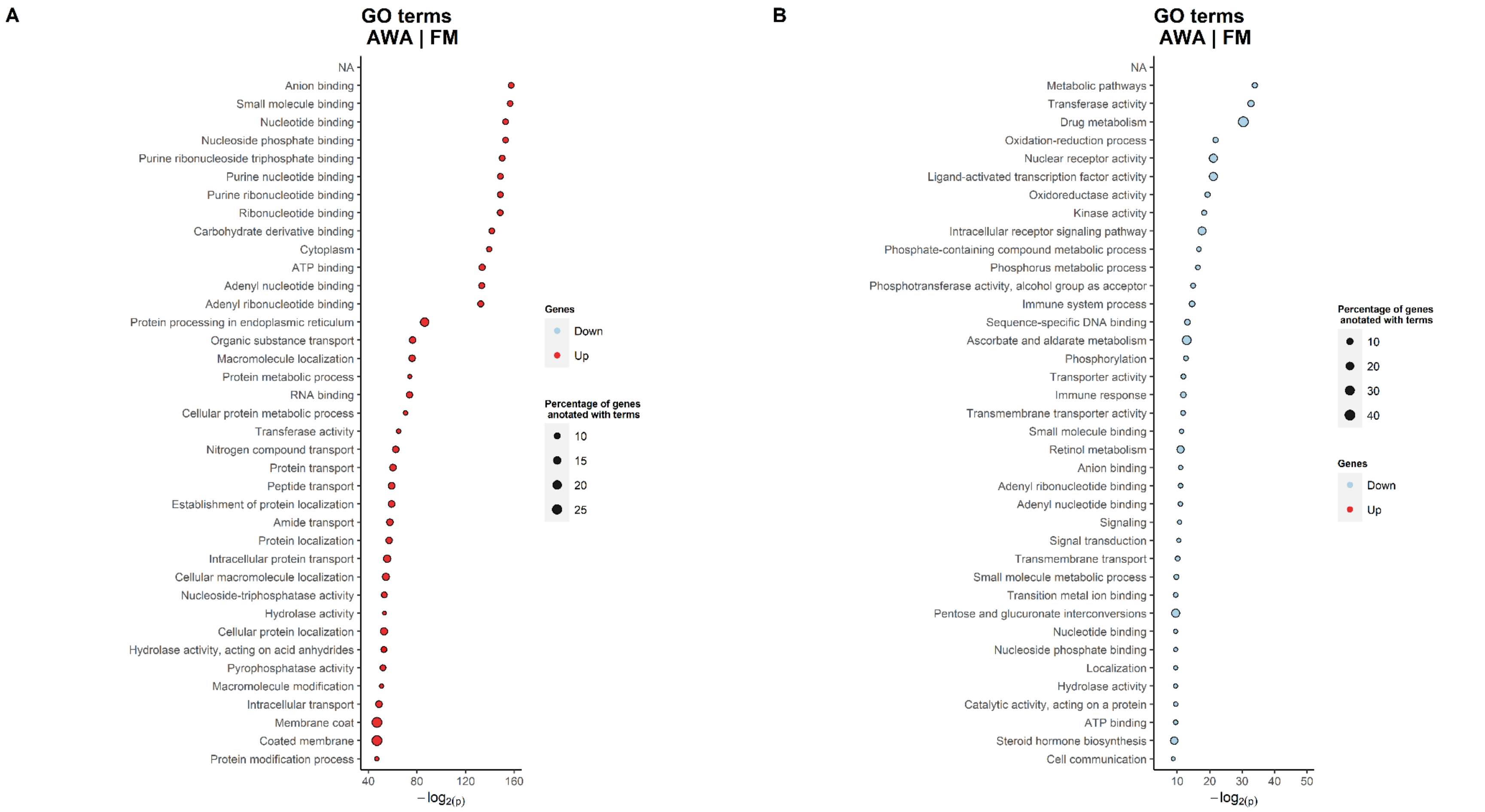

FIGURE S4 Significantly enriched gene ontology (GO) terms (minGSSize = 3) in distal intestine of Atlantic salmon smolts fed AWA diet compared with fish fed FM diet. The list is ordered by decreasing Enrichment Score (-log2(P)). A. Up, upregulated (in red); B. Down, down-regulated (in light blue). The diets were: FM – fishmeal-based; and diet containing 300 g/kg SBM and 100 g/kg of IWA – autolyzed *Wickerhamomyces anomalus*. The top 40 up-regulated and down-regulated genes are presented.
